# Traumatic Pubic-type Anterior Dislocation of the Hip with an Ipsilateral Greater Trochanter Fracture: Case Report and Review of Literature

**DOI:** 10.7759/cureus.3287

**Published:** 2018-09-11

**Authors:** Rajkumar Selvanayagam, Vivek Tiwari, Saubhik Das, Vivek Trikha

**Affiliations:** 1 Department of Orthopaedics, All India Institute of Medical Sciences, Bhopal, IND; 2 Department of Orthopaedics, All India Institute of Medical Sciences, New Delhi, IND

**Keywords:** anterior hip dislocation, pubic dislocation, greater trochanter fracture, fracture dislocation of hip, hip trauma, osteonecrosis

## Abstract

Due to the inherent stability of the hip joint, hip dislocations constitute a relatively small proportion of all the traumatic dislocations encountered in the emergency department. Among them, the anterior type of hip dislocation is less common than the posterior type of dislocation. Anterior dislocations are usually associated with an injury to other, nearby structures like the acetabulum and femoral head. An ipsilateral greater trochanter fracture with anterior hip dislocation is very sparsely reported in the literature. We report the case of a pubic type of anterior hip dislocation associated with a concomitant ipsilateral greater trochanter fracture. The joint was reduced promptly with traction-countertraction under sedation, and the associated fracture was subsequently fixed with two 6.5 mm partially threaded, cannulated, cancellous screws. The patient was symptom-free at the last follow-up of one year with a full range of hip joint motion, and without any evidence of osteonecrosis or osteoarthritis. The mechanism of greater trochanter fracture in such injuries and its management has been discussed.

## Introduction

Hip joint has inherent stability due to bony as well as soft-tissue constraints including a deep socket of the ball-and-socket joint, thick muscular envelope of gluteal muscles, and strong supporting ligaments. Nevertheless, high-velocity trauma to the lower limbs is not infrequently associated with posterior hip dislocations, especially with dash-board injuries. Anterior type of hip dislocations are less common constituting around 15% of all hip dislocations [1-2]. Such dislocations are often associated with injury to the nearby structures including fractures of the acetabulum and femoral head [2-4]. However, concomitant ipsilateral greater trochanter fractures are sparsely reported in the literature. Furthermore, pubic-type of anterior hip dislocations are extremely rare. We report an unusual case of pubic-type anterosuperior hip dislocation with ipsilateral greater trochanter fracture and subsequently discuss the probable mechanism of injury and its management.

## Case presentation

A 28-year-old man was brought to our emergency department with severe right hip pain with deformity following a high-velocity motor vehicle accident due to the collision of his car with a truck. He was conscious with Glasgow Coma Scale 15 with stable vital parameters. After an initial assessment of the patient, according to the Advanced Trauma Life Support protocol, a secondary survey revealed his right hip in an abducted and externally rotated position with shortening of the right lower limb. On further examination, the femoral head was palpable in the ipsilateral inguinal region. There was no external bleeding wound and no associated distal neurovascular deficit.

**Figure 1:**
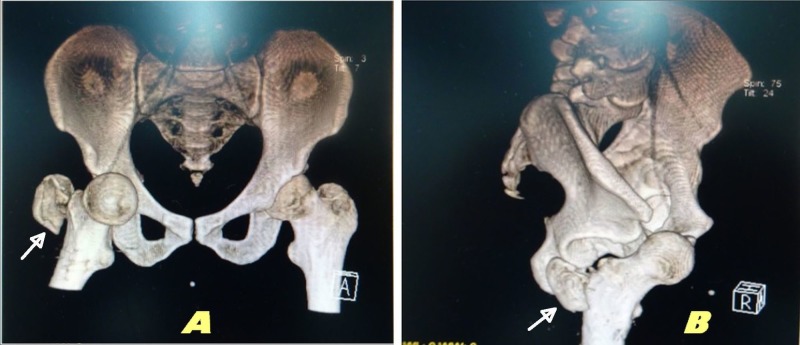
Computed tomography (CT) scan of the pelvis with three-dimensional (3D) reconstruction from the anterior aspect (A) and right lateral aspect (B) Pubic-type anterior dislocation of the right hip with ipsilateral displaced greater trochanter fracture (white arrows).

The routine imaging investigations and examination ruled out any head, cervical, thoracic or abdominal injury. Plain radiograph of the pelvis with both hips revealed a pubic-type anterior dislocation of the right hip with ipsilateral greater trochanter fracture. A computed tomography (CT) scan of right hip was also ordered to look for any associated acetabular fracture, intra-articular fragment, occult femur neck or intertrochanteric fracture; it ruled out any associated injury and confirmed anterior hip dislocation with ipsilateral greater trochanter fracture (Figure 1).

**Figure 2:**
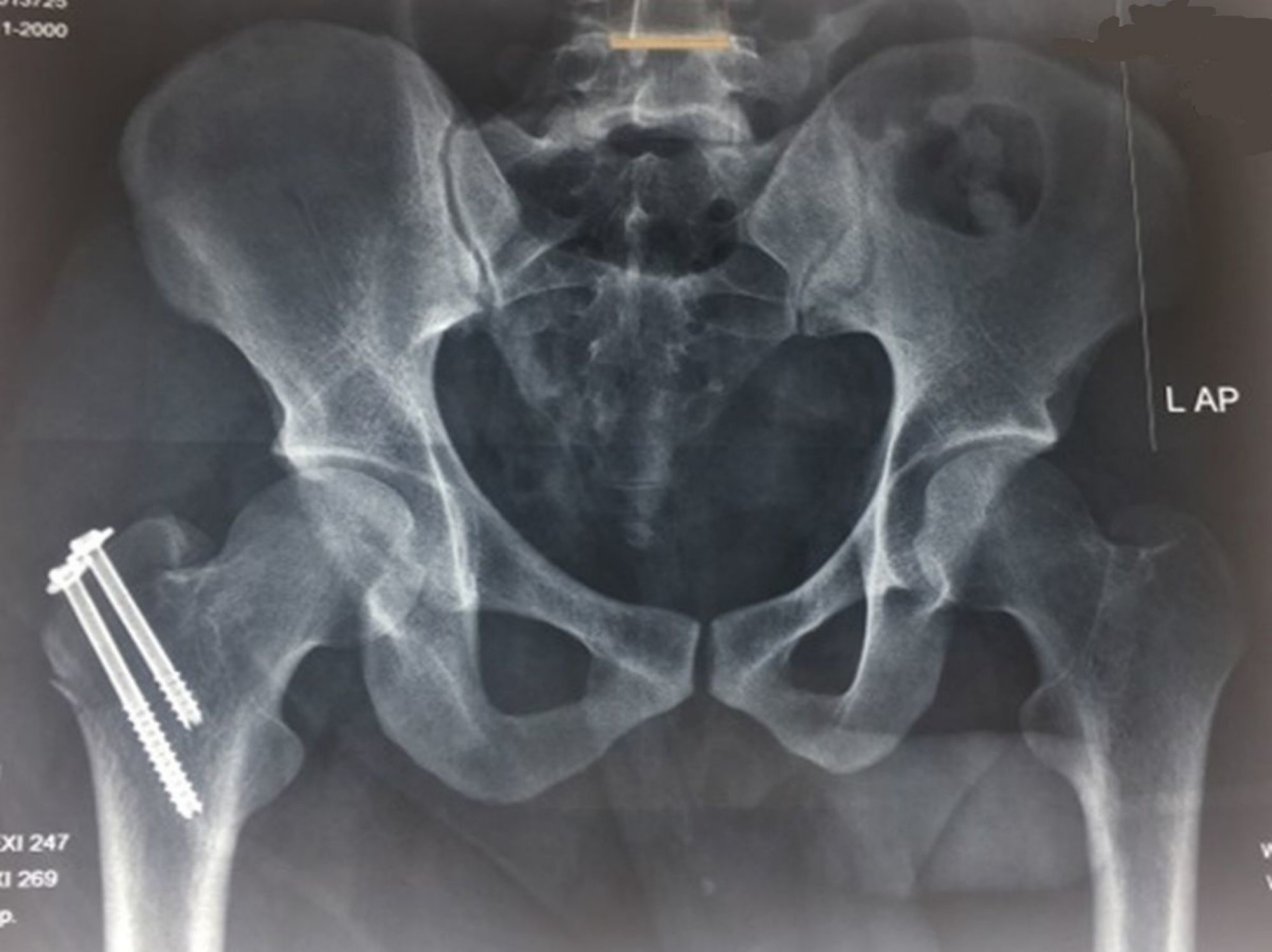
Post-operative plain radiograph of the pelvis with bilateral hips – anteroposterior view Fixation of greater trochanter fracture with two 6.5 mm cannulated cancellous screws with washer, along with congruent reduction of the right hip joint.

We performed a closed reduction of the dislocation under sedation within two hours of the accident in the emergency department. The patient was positioned supine and the reduction involved the collective effort of four persons; the pelvis was stabilized by one resident, another person pushed the femoral head into the acetabulum by direct palm pressure while the other two gave continuous axial traction in the extended position followed by flexion and internal rotation. A snap sound suggesting relocation of the femoral head followed this reduction maneuver. The post-reduction plain radiograph of the pelvis showed a congruent reduction of the hip joint along with a displaced fracture of the greater trochanter. Subsequently, open reduction and internal fixation were planned for the fracture in the elective theatre the next day. Under C-arm guidance in the left lateral position, the fracture was reduced and two 6.5 mm partially threaded cannulated cancellous screws were inserted through a mini-incision under spinal anaesthesia (Figure 2).

The postoperative period was unremarkable and the patient was kept non-weight bearing on the affected limb for two weeks followed by partial-weight bearing over the next two weeks. The patient was allowed full-weight bearing after one month. At the last follow-up of one year, the patient was asymptomatic with a full range of active and passive right hip joint motion. There was no evidence of hip osteoarthritis or osteonecrosis of the femoral head.

## Discussion

Hip dislocation is a relatively infrequent injury pattern as it requires significant force to occur owing to the inherent stability of the joint. Among hip dislocations, posterior dislocation is much more commonly seen than the anterior variety. Anterior dislocations result from forced abduction and external rotation moment to the hip. They have further been classified into two types – pubic and obturator [3]. In the more common flexion or obturator type, the femoral head dislocates inferiorly, and the limb is abducted and externally rotated with femoral head palpable near the obturator foramen. In the extension or pubic type, the hip dislocates anteriorly and superiorly. The limb appears short, and the thigh is extended and externally rotated with the femoral head palpable in the groin, as seen in our case. The pubic-type of anterior hip dislocation is not commonly reported in the literature. Though obturator type of dislocation can be easily diagnosed on plain anteroposterior radiographs, pubic type is often misdiagnosed as posterior hip dislocation [5]. The position of lesser trochanter serves as a useful guide in such situations. In the anterosuperior dislocation, the hip is in external rotation attitude with the lesser trochanter being excessively prominent, whereas in posterior dislocation, the hip is internally rotated, with the trochanter being less visible or hidden [5].

Anterior hip dislocations are reported to be associated with concomitant acetabular or femoral neck injuries, which were ruled out in our case on three-dimensional (3D) CT scans. However, isolated greater trochanter fracture is very rarely described with anterior dislocations, especially with the pubic type. Maruoka reported a case of pubic dislocation of the femoral head with ipsilateral greater trochanter fracture, managed with closed reduction and a hip spica cast for six weeks [6]. Granahan described another case of traumatic pubic dislocation with greater trochanter fracture along with concomitant short segment dissection of the ipsilateral external iliac artery; after reduction of dislocation, the fracture and vessel injury were managed conservatively [7]. There was no distal neurovascular injury in our case. Atchi reported an open pubic-type anterior dislocation of hip along with ipsilateral greater trochanter fracture and contralateral iliac wing fracture following a high-velocity motor vehicle accident. After reduction of the hip joint and wound debridement, the authors managed the fractures conservatively owing to non-availability of implants, and subsequently had poor results with hip osteoarthritis and femoral head osteonecrosis at one-year follow-up [8].

In another case of open pubic-type anterior hip dislocation with greater trochanter fracture, the dislocation was reduced along with wound debridement and the fracture was treated conservatively. The wound healed well without the need for redebridement; however, at one-year follow-up, the patient had a significantly decreased range of hip movements despite the lack of femoral head osteonecrosis or infection, owing to the presence of superior labral lesion [9]. Freitas et al. reported another open pubic-type anterior hip dislocation with ipsilateral greater trochanter fracture, in which the fracture was fixed with two 6.5 mm screws following open reduction of the hip and wound debridement. The wound condition required vacuum-assisted closure followed by skin grafting, and at one-year follow-up, the patient had good mobility with a Harris hip score of 93 points [10]. As compared to the aforementioned cases, our patient had a closed injury without any associated vascular insult. Interestingly, the trochanteric fracture was managed conservatively in the majority of the reported cases. However, as in the case by Freitas et al., operative fixation of the greater trochanter was done in our case as it helps in providing early functional recovery.

The mechanism through which greater trochanter fracture occurs along with anterior hip dislocation without acetabular wall and femoral head injury is ill-defined. Maruoka et al. discussed the probable mechanism of greater trochanter fracture in such injuries by simulating pubic-type anterior hip dislocation in a cadaveric specimen [6]. When the impact of injury is such that the hip becomes extended, slightly externally rotated and maximally abducted, the greater trochanter impinges on the ilium. The resulting blow of the ilium on the greater trochanter results in its fracture. Simultaneously, the femoral head also gets levered out of the acetabulum resulting in pubic-type anterior hip dislocation. The pull of the hip external rotators and abductors cause the fracture fragment to get displaced posteriorly and laterally. In order to maintain the abductor length and avoid subsequent lurch, and also to provide early mobilization, we recommend open reduction and operative fixation of the greater trochanter fracture in these cases.

## Conclusions

We discussed a patient with a unique pattern of injury consisting of pubic-type anterior hip dislocation with ipsilateral greater trochanter fracture, which does not fit into any current classification system described for hip fracture-dislocation injuries. We recommend that such classification system is reviewed to encompass these unusual patterns of injury as well. The dislocation was promptly reduced and the associated fracture was fixed with two screws to provide early functional recovery. The above treatment resulted in a full range of hip movements without any evidence of osteonecrosis or osteoarthritis at the end of one year.
